# Global Health Equity Requires Global Equity

**DOI:** 10.1089/heq.2022.0169

**Published:** 2023-03-21

**Authors:** Nason Maani, Salma M. Abdalla, Catherine K. Ettman, Lily Parsey, Emma Rhule, Pascale Allotey, Sandro Galea

**Affiliations:** ^1^ Global Health Policy Unit, School of Social and Political Science, University of Edinburgh, Edinburgh, United Kingdom.; ^2^ Rockefeller Foundation/Boston University Commission on Data, Determinants and Decision-making, Boston, Massachusetts, USA.; ^3^ Boston University School of Public Health, Boston, USA.; ^4^ Johns Hopkins Bloomberg School of Public Health, Baltimore, Maryland, USA.; ^5^ International Longevity Centre UK (ILC), London, United Kingdom.; ^6^ United Nations University International Institute for Global Health, Kuala Lumpur, Malaysia.

**Keywords:** social determinants of health, health inequality, decision-making, global health

## Abstract

Many global health challenges are characterized by the inequitable patterning of their health and economic consequences, which are etched along the lines of pre-existing inequalities in resources, power, and opportunity. These links require us to reconsider how we define global health equity, and what we consider as most consequential in its pursuit. In this article, we discuss the extent to which improving underlying global equity is an essential prerequisite to global health equity. We conclude that if we are to improve global health equity, there is a need to focus more on foundational—rather than proximal—causes of ill health and propose ways in which this can be achieved.

## Introduction

The disparities in health outcomes revealed and exacerbated during the recent pandemic^1^ are the latest manifestation of longstanding global health inequity.^2^ Global health equity is often discussed in conversations on development as a policy goal, a field of research, an ethical imperative, a health sciences discipline, and is even the overarching theme of a university. However, as an aspiration, what might global health equity truly mean? To answer this, we revisit each concept within this term and discuss possible implications for research and policy.

A global perspective require a lens through which the outcomes for all are equally valued, regardless of national borders, gender, age, race or ethnicity, tribe, class, ability, sexual orientation, or income or other social and economic stratifiers. At core, a global lens requires us to assess need and priorities at a supranational level. Taking such a lens is akin to conceptualizing the earth as a single country, in which all citizens are viewed as interconnected and interdependent, having the same fundamental rights. With this lens in mind, we turn to health. The World Health Organization (WHO) defines health as “…a state of complete physical, mental, and social wellbeing and not merely the absence of disease or infirmity.”

It is well established that health gaps are to a large degree shaped by inequalities in our social and physical environments, as painfully laid bare by the pandemic.^3^ Health, then, is about much more than health care, serving largely as a reflection of the upstream distribution of power, opportunity, and resources. Equity describes the just and fair allocation of resources according to need. It describes the absence of avoidable differences among different groups of people, whether we define them by their geographic location, rurality, economic status, or social standing. In the context of health, it refers to the allocation of resources according to need, in a way that preventable differences in health outcomes are minimized, and access is fair.^4^

With these definitions in mind, considering the broader drivers of health globally, it is clear that equity in the context of health requires equity in the political, social, and economic conditions that generate health. For this reason, it has been argued that global health is not merely an academic discipline but “a collection of problems [that] …turn on the quest for equity.”^5^ Yet, as a metaknowledge analysis of global health literature recently found, global health scholarship is dominated by a focus on specific areas such as infectious diseases, disease surveillance, maternal and child health, and health systems governance.^6^ In other words, while the roots of health lie in the “upstream” conditions that generate health, and conceptually, the parameters of global health equity are defined in broad terms, the practice of global health scholarship focuses overwhelmingly on the “downstream” health consequences of inequity.

## What Would Achieving Global Health Equity Mean?

### A world where health itself is equitably distributed between countries and regions

While much recent attention has been given to the COVID-19 pandemic, significant, long-standing gaps remained in life expectancy between countries, despite a rapid narrowing in the last 50 years.^7^ The WHO Africa region continued to experience a disproportionately high disease burden in terms of disability-adjusted life years, primarily driven by neonatal conditions and preventable infectious diseases such as lower respiratory infections, malaria, diarrheal diseases, HIV/AIDS, and tuberculosis.^8^ Achieving global health equity would mean a world in which this disproportionate and avoidable disease burden, which primarily affects the very young and the very poor, would be eliminated.

Yet, doing so would require engagement far beyond the medical, even though much focus remains on health security and universal health coverage.^9^ While medical innovation has played an important role particularly in the context of infectious diseases, at the global level, improvements in population health have been largely due to the improvements in living conditions, employment, and education associated with economic development. A large resource disparity between the global north and global south represents a political and economic power imbalance that risks obstructing multilateralism and the protection and growth of global public goods.

This manifests in many ways, including trade objections by high-income countries preventing national public health regulations in low- and middle-income countries,^10^ and during the pandemic, in vaccine and personal protective equipment stockpiling by high-income countries at the expense of low- and middle-income countries,^11^ or in challenges reaching agreement and delivering on loss and damage funds related to global warming.^12^ In other words, between-country health inequalities are, in large part, a consequence of between-country power and resource inequalities. However, even if such foundational inequalities between countries were to be addressed, these would still not be sufficient to achieve health equity globally, due to the profound inequalities that exist within countries, in both the global north and the global south.^13^

### A world where health is not inequitably distributed within countries

Taking a truly global lens, in which all citizens are viewed as interconnected, interdependent, and of equal value, requires us to consider not only the inequitable distribution of health across countries, but also within countries, regions, cities, and neighborhoods.^14^ For example, in the United States in 2016, men aged 40 years in the highest 1% of earners had an expected age of death of 87.3 years, 14.6 years longer than those in the bottom 1% of earners. The equivalent gap for women was 10.1 years.^15^ Life expectancy at birth in India is 71.6 years in the wealthiest quintile of men, decreasing to 63.2 in the poorest quintile overall, but these gaps are larger in urban households (9.1 years) compared to rural households (7.5 years).^16^

Beyond urban/rural divides, regional differences reflecting resource and opportunity gaps can lead to significant within-country variance. This means that in many countries in both the global north and the global south, significant proportions of the population are “left behind,” experiencing shorter lives in poorer health, often along racial and ethnic divides.^17,18^ As with between country health gaps, these within-country gaps are a consequence of wider socioeconomic inequalities, of often long-standing and entrenched gaps in access to power, opportunity, and resources. It has been argued that in global health, there is limited reflection on how movements such as decolonization might engage with such axes of power and disadvantage that reside within national borders, yet, clearly there is a need for an intersectional understanding that encompasses the effects of deep cultural or socioeconomic divides, such as casteism.^19^

It is, however, important to understand differences in within- and between-country health inequalities, as their causes, measurement, and solutions vary. For example, trade agreements that widen access to the global economy may reduce between-country health inequalities, but prove a catalyst for the widening of health inequalities within a country. In high-income countries, the benefits of such trade may in some cases disproportionately fall to a wealthier and more educated subsection of the population, while the risks, in the form of job loss, outsourcing, or reduced employment rights, fall disproportionately on low-skill workers.^20^ Persistent within-country inequalities may also undermine public support for trade agreements^21^ or investing in international development.

Understanding and mitigating these trade-offs as a foundation of global health equity is critical as both between- and within-country health gaps pose threats to health and global prosperity.^22^ When considering between-country or within-country differences, it is clear that health inequities arise due to wider inequalities in the allocation of power, resources, and opportunity. It is through that lens that we must examine and overcome barriers to global health equity in its broadest sense.^19^ In considering the foundational drivers behind reductions in global health inequities to date, we identify three main areas that have inhibited progress toward global health equity.

### A narrow emphasis on what shapes health, available to a few

In contrast to the broad definition of global health equity, global health research in practice reflects a narrow emphasis on particular health outcomes in particular settings, reflecting the complex, and colonial history of global health, its emergence from international health, and the power assymetries inherent in its funding, leadership, and priorities.^23^ A recent meta-knowledge analysis found that global health research remains focused largely on infectious diseases and health care systems, rather than important priority areas such as urbanization, climate change, antimicrobial resistance, or income inequality.^6^

### A lack of focus on the foundational causes of health, which are highly inequitable in their distribution

A focus on the downstream and biomedical solutions to global health equity has also led to a lack of focus on the social determinants of health, the physical and social environments in which we live, even though these are highly inequitable in their distribution, and contribute disproportionately to health inequity within and between countries.^24,25^ These divides reflect fundamental divides in power and opportunity, accompanied by differences in soft power and prestige, supported by longstanding values and norms. Beyond hard barriers, these divides contribute to feelings of stigma, marginalization, dispossession, and diminishment in engagement with authorities, including health authorities, exacerbating divides in both health and human capital.^26,27^

While a renewed focus on the global value of health security in the wake of the pandemic is welcome, and universal health coverage unquestionably an important goal, the central importance of the social determinants of health means that these initiatives will in themselves not lead to health equity. If the postpandemic aspiration is, in the face of overwhelming evidence of global interconnectedness, to operate on a “no-one is safe until everyone is safe” principle, extremes of wealth and poverty, of power and dependency, between and within nations, must be a central concern in any health conversation.

### A lack of focus on the causes of inequitable distributions

Considering the roots that underpin health inequity, achieving true global health equity requires us to address the broader, deeper power and economic inequities that ultimately underpin these challenges, within and between countries.^28^ This lens requires us to consider global values (for example, see Box 1) that can guide both international and national levels of action, since both are fundamental components of global health equity. It also requires us to consider all decisions as health equity decisions, since health is affected by decisions across government departments.

**Box 1.** Values that could guide global equity priorities

• Equity as a critical component of resilience and disease prevention
• Reducing health inequity necessitates that we reduce underlying within- and between-country inequities
• The urgency of current public health crises cannot distract from the need to address the foundational causes of these crises
• All decisions that affect equity affect health

In this sense, global health has the responsibility to be self-critical in nuanced and consequential ways. Health spending makes up a substantial portion of all philanthropic development aid, much of which is focused on vertical funding, targeted at specific disease outcomes.^29^ The often fragmented, inadequately tracked, typically downstream nature of global health finance is at odds with the foundational importance of the social determinants of health, and the role of wealth, power, and opportunity in building more healthy and resilient individuals, communities, and nations.

#### A way forward

The highly inequitable effects of the COVID-19 pandemic, of global warming, or the global reverberations of energy and food shortages triggered by the war in Ukraine serve to demonstrate the extent to which pre-existing inequities within- and between-countries have long undermined health and prosperity. While rightly focusing on the acute needs of health crises and gaps, striving for global health equity requires us more explicitly tie health goals, data, decisions, and accountability to its upstream determinants. We can in each case focus only on targeted aid to ameliorate the worst downstream effects of each crisis, or acknowledge that inequity, within and between countries, is at the core of the majority of such effects.

It requires us to engage much more proactively with the equity trade-offs of wider decisions regarding trade, employment, human rights, and international development, as interventions that may have enormous downstream health impacts. We end with three reflections for researchers, practitioners, and policy-makers centrally interested in improving global health equity.

First, if within- and between-country health inequalities are in the main a reflection of wider injustices, then our understanding of data most relevant to global health must widen to encompass the social, political, and commercial determinants of health.^30,31^ In our focus on individual disease risk factors and outcomes, we risk overreliance on external and biased perspectives on the health needs of the most margalized and disempowered, becoming, what Anthony McMichael has termed, “prisoners of the proximate,” to the detriment of the most consequential health improvements.^32^ If achieving global health equity is impossible without global equity, then we must become increasingly familiar with, and involved in, the processes, disciplines, and data that drive wider equity.

Second, a broader understanding of both between- and within-country inequalities and the highly context specific cultural and historical backgrounds to such inequalities requires us to engage more deeply with what a more diverse workforce, in research and in practice, would constitute. This reflects an increasing understanding of the importance of intersectionality in both representation and capacity building in ways that more consciously acknowledge both within- and between-country power imbalances along socioeconomic, racial, ethnic, or gender lines.^13,33^

Third, there is a need to better tell the story of health equity as a consequence of wider equity. In recent years, health professionals, often primarily dealing with the downstream consequences of social determinants, have lent their voices to the importance of social determinants of health, and had to become increasingly familiar with upstream forces that shape health outside the clinic.^34,35^ So, the global health community too must become more deeply engaged with wider processes of social change, in acknowledgment of the limitations of downstream actions to improve health equity.

## Abbreviation Used

WHO: World Health Organization
